# Supplementary material for: Chromothripsis during telomere crisis is independent of NHEJ, and consistent with a replicative origin
Source: Genome Res. 2019 May;29(5):737–49. doi: 10.1101/gr.240705.118 (PMC6499312; doi:10.1101/gr.240705.118)
Supplement: Supplemental Material [file supp_gr.240705.118_Supplemental_file_1.zip › contigs/annotated_contigs/DB86/contig.2.DB86_length_648_mean_cov_5.80864197531.docx]

**DB86_length_648_mean_cov_5.80864197531**

```
TAACATATATAGCATAATTTTTAACATTATATGGATCTCTACATATAGAGAAAAAGAGACATTATACATTGTGTGTAGATGGATAGATA
 >chrX:7405328-7405601 - E=4e-141
GATAGATAGATAGATAGATAGATAGATAGATAGATAGATAGATAGAAAGAAAATTTCAATTTAAAGCTCAACTTTTCCCAATTACCTTT

TTCAAAAAGTTCTCAGTCTAAAATTCAACAATCTTTGCATGCAGCTGAATCAAGAAGAGGTTTAATCTCCCATTATTCTCTGTACACAG

AACCAATATCTTTT|AT|CTTTGTAGGGAAATGGATGAAGCTGGAAACCATCATTCTCAGCAAACTATCACAAGGACAAAAAAACCAAA
                 >chrX:7369534-7369859 - E=8e-184
CACCATATGTTCTCACTCATAGGTGGGAACTGAACAATGAGAACAAACGGACACAGGAAGGGGAACATCACACACCAGGGCCTGTTGTG

GGGTGGGAGGAGGGGGGAGGGATAGCATTAGGAGATAATACCTAATGTTAAATGACAAGTTAATGGGTGCAGCACACCAATATGGCACA

TGTATACATATATAACAAACCTGCATGTTGTGCACATGTACCCTAAAACTTAAAATATAATAAAAAAAAAAAAAGA|TGCCAATTTAAG

ACGGCTCATTTCTTTTTTATCAGTTGCC
```
